# Nutraceuticals and Pharmaceuticals from Marine Fish and Invertebrates

**DOI:** 10.3390/md19070401

**Published:** 2021-07-20

**Authors:** Vida Šimat

**Affiliations:** University Department of Marine Studies, University of Split, Ruđera Boškovića 37, 21000 Split, Croatia; vida@unist.hr

The word nutraceutical is coined from two words, “nutrition” and “pharmaceutical”, describing natural sources of molecules or substances that have physiological benefit, and the ability to protect human health and well-being and prevent chronic diseases [1]. These molecules can be incorporated into foods, or used as drugs or supplements. The high nutritional value of fish and marine invertebrates makes them an ideal and essential food choice that may contribute to the imbalance in the lifestyle and diet in developed countries that has been causing a rise in chronic diseases, such as obesity, diabetes, hypertension, and hyperlipidemia, and other lifestyle-related diseases [2]. There are various ingredients found in fish and fish by-products that act as nutraceuticals. The most distinctive are long-chain polyunsaturated fatty acids and other lipid components, protein hydrolysates and peptides, minerals and vitamins, gelatin, and collagen. Among many beneficial effects, they show a therapeutic effect against cardiovascular diseases, hypertension, asthma, inflammatory bowel disease, rheumatoid arthritis, osteoporosis, and even cancer [3].

The diversity of organisms in the marine ecosystem presents a reservoir of a huge variety of natural compounds, and every year novel marine molecules are being identified, as well as their biological potential, allowing the use of these compounds in functional food application, as an ingredient in commercial products and the development of new nutraceuticals, as therapeutic agents, and in pharmaceuticals. Marine fish are traditionally accepted as a healthy choice of food, but recent developments in the field of nutraceuticals and functional foods are revealing that their value goes beyond nutritive, as they supply our diet with molecules that play a major therapeutic role against human diseases or in their prevention. In recent times, even the by-products from the seafood industry have been described as a source of valuable compounds such as enzymes, peptides, and collagen that may contribute to human health or be used in the design of new drugs [4]. On the other hand, marine invertebrates present a large and diverse group of organisms that have a broad geographic distribution and include organisms such as sponges, cnidarians, marine worms, lophophorates, mollusks, arthropods, echinoderms, and the hemichordates. They have been studied for decades, since many of them produce metabolites with strong biological potential. Among them, sponges have been identified as an outstanding source of biologically active compounds. Actually, in the last 30 years of research, out of 9812 biologically active marine metabolites from invertebrates, almost 50 percent can be traced to sponges [5]. A potential candidate for the functional food development, nutraceutical, and pharmaceutical sectors is the sea cucumber. Its potential therapeutic benefits are related to its impressive nutritional profile, which includes collagen, omega-3 and omega-6 fatty acids, vitamins A, B1 (thiamine), B2 (riboflavin), and B3 (niacin), and minerals, but also compounds such as saponins, sulfated polysaccharides, phenolics, peptides, and others [6]. Despite their ability to produce bioactive secondary metabolites with nutraceutical and pharmaceutical potential, only a small share of the invertebrate’s metabolites has been chemically characterized and investigated. Additionally, some compounds first isolated from invertebrates have been produced by their symbiotic microbes [7].

This Special Issue of *Marine Drugs*, “Nutraceuticals and Pharmaceuticals from Marine Fish and Invertebrates” consists of four articles and three reviews devoted to recent advances in the study of the bioactive compounds from marine fish and invertebrates and their by-products, including enzymes, pigments, peptides, proteins, essential fatty acids (particularly eicosapentaenoic and docosahexaenoic acid), vitamins, and minerals, as well as their characterization and biological activity. The research papers in this Special Issue present some new molecules that have wide possibilities for application, from contributions in cancer therapy and chemo-preventive effects to bactericidal effects.

Abdelhameed et al. described three new cerebrosides and cholesterol sulfate isolated from the sea cucumber *Holothuria spinifera* from the Red Sea, with the potential for becoming new anticancer drug candidates. The new compounds demonstrated promising antitumor activity in the breast carcinoma cell line MCF-7 in comparison to the control drug doxorubicin [8]. The paper of Jiang et al. demonstrated the ameliorating effect of pentadecapeptide from *Cyclina sinensisat* (SCSP) against cyclophosphamide (CTX), a widely used antitumor drug affecting cell morphology and organ function, that causes nephrotoxicity and kidney tissue damage. In a mice model, SCSP was shown to be effective for inhibition of the activities of antioxidant enzymes and markers in the kidney and reduction in inflammation. Furthermore, SCSP showed the ability to restore the protein levels of the members of the NF-κB and apoptotic signaling pathways in the kidney, and may be used as a therapeutic adjuvant to ameliorate CTX-induced nephrotoxicity [9].

Another new molecule, a *Ciona* molecule against microbes-A24 (CiMAM), was described by Lee et al. [10] as an alternative to the antimicrobial peptide that was isolated from the marine chordate *Ciona intestinalis*, using a microorganism generally recognized as safe (GRAS), *Bacillus subtilis*, as a host cell. The CiMAM maintained its bactericidal activity, even in a very high salt and ion environment, making the application of recombinant CiMAM-expressing transgenic *B. subtilis* strains a promising candidate to protect marine fish and shellfish from halophilic bacterial infection.

The by-products of processing marine organisms are being recognized as raw materials of significant value, and their valorization is becoming an important way of assuring sustainable, profitable, and ecologically acceptable seafood production. In the paper of Wang et al., one of the most acceptable methods of by-product valorization was investigated, the use of by-products to produce high-value compounds with bioactive properties [11]. The authors prepared and characterized (amino acid sequence and molecular mass) six antioxidant peptides from collagen hydrolysate of redlip croaker (*Pseudosciaena polyactis*) scales, showing their potential to be used as antioxidants in nutraceutical and pharmaceutical products, especially their cytoprotective effects on H_2_O_2_-damaged HepG2 cells. 

The reviews in this Special Issue describe the recent advances and state-of-the-art knowledge in the field of marine-based nutraceuticals, their health benefits, and potential application in value-added functional food products. The recent studies of functional seafood compounds (chitin and chitosan, pigments from algae, fish lipids and omega-3 fatty acids, essential amino acids and bioactive proteins/peptides, polysaccharides, phenolic compounds, and minerals) and their potential use as nutraceuticals [12], as well as their therapeutic benefits and possible application in the treatment of chronic diseases and conditions [2], will contribute to the global nutraceutical management and market through the development of new functional food, beverage, and supplement products. 

Finally, this Special Issue publishes an overview of 43 years of research of chemical structures and biologically active metabolites from the marine sponges of the genus *Petrosiai*, indicating a need to include research on the symbiotic relationships between marine sponges and microorganisms and the discovery of novel genes involved in biosynthesis to provide potential bioengineering applications in future research perspectives [5]. 

## References

[1] Nasri H., Baradaran A., Shirzad H., Rafieian-Kopaei M. (2014). New concepts in nutraceuticals as alternative for phaarmaceuticals. Int. J. Prev. Med..

[2] Ashraf S.A., Adnan M., Patel M., Siddiqui A.J., Sachidanandan M., Snoussi M., Hadi S. (2020). Fish-based bioactives as potent nutraceuticals: Exploring the therapeutic perspective of sustainable food from the sea. Mar. Drugs.

[3] Guo C.-H., Hsia S., Chung C.-H., Lin Y.-C., Shih M.-Y., Chen P.-C., Hsu G.-S.W., Fan C.-T., Peng C.-L. (2021). combination of fish oil and selenium enhances anticancer efficacy and targets multiple signaling pathways in anti-VEGF agent treated-TNBC tumor-bearing mice. Mar. Drugs.

[4] Al Khawli F., Pateiro M., Domínguez R., Lorenzo J.M., Gullón P., Kousoulaki K., Ferrer E., Berrada H., Barba F.J. (2019). Innovative green technologies of intensification for valorization of seafood and their by-products. Mar. Drugs.

[5] Lee Y.-J., Cho Y., Tran H.N.K. (2021). Secondary metabolites from the marine sponges of the genus petrosia: A literature review of 43 years of research. Mar. Drugs.

[6] Hossain A., Dave D., Shahidi F. (2020). Northern sea cucumber (*Cucumaria frondosa*): A potential candidate for functional food, nutraceutical, and pharmaceutical sector. Mar. Drugs.

[7] Dou X., Dong B. (2019). Origins and bioactivities of natural compounds derived from marine ascidians and their symbionts. Mar. Drugs.

[8] Abdelhameed R.F.A., Eltamany E.E., Hal D.M., Ibrahim A.K., Aboul Magd A.M., Al-Warhi T., Youssif K.A., Abd El-kader A.M., Hassanean H.A., Fayez S. (2020). New cytotoxic cerebrosides from the red sea cucumber holothuria spinifera supported by in-silico studies. Mar. Drugs.

[9] Jiang X., Ren Z., Zhao B., Zhou S., Ying X., Tang Y. (2020). Ameliorating effect of pentadecapeptide derived from cyclina sinensis on cyclophosphamide-induced nephrotoxicity. Mar. Drugs.

[10] Lee B.-C., Tsai J.-C., Lin C.-Y., Hung C.-W., Sheu J.-C., Tsai H.-J. (2021). Using bacillus subtilis as a host cell to express an antimicrobial peptide from the marine chordate ciona intestinalis. Mar. Drugs.

[11] Wang W.-Y., Zhao Y.-Q., Zhao G.-X., Chi C.-F., Wang B. (2020). Antioxidant peptides from collagen hydrolysate of redlip croaker (*Pseudosciaena polyactis*) scales: Preparation, characterization, and cytoprotective effects on H2O2-damaged HepG2 cells. Mar. Drugs.

[12] Šimat V., Elabed N., Kulawik P., Ceylan Z., Jamroz E., Yazgan H., Čagalj M., Regenstein J.M., Özogul F. (2020). Recent advances in marine-based nutraceuticals and their health benefits. Mar. Drugs.

